# Perceived human-robot relational structures: a pictogram-based questionnaire to assess ideal and assumed relationships

**DOI:** 10.1038/s41598-026-47643-y

**Published:** 2026-04-16

**Authors:** Tomonori Kubota, Takahisa Uchida, Hideyuki Takahashi, Midori Ban, Hiroshi Ishiguro

**Affiliations:** 1 Graduate School of Engineering, Nagoya University, Nagoya, 464-8601 Japan; 2 Graduate School of Engineering Science, The University of Osaka, Toyonaka, 560-8531 Japan; 3 Faculty of Science and Engineering, Otemon Gakuin University, Ibaraki, 567-8502 Japan

**Keywords:** Engineering, Psychology, Psychology

## Abstract

Evaluations in human-robot interaction (HRI) have traditionally focused on surface-level impressions such as likability and anthropomorphism, often neglecting the underlying relational structures that define mutual contributions and shared goals. Understanding these relational dynamics is crucial for fostering effective and sustainable human-robot collaborations. Here, we developed and validated a novel pictogram-based questionnaire grounded in social psychology to capture relational structures, conducting three scenario-based online surveys. We examined ideal and assumed relational structures in human-human and human-robot contexts across varying robot appearances, roles, and utterances. We distinguish between ideal relational structures (what users would like to establish; Surveys A and B) and assumed (expected) relational structures (what users believe would emerge under the described conditions; Survey C). Our results reveal that ideal relational structures vary by human relationship type, and that a robot’s communicative behavior significantly influences perceived relational structures, often more than physical appearance. These findings suggest that assessing relational perceptions can inform the design of robots better aligned with user expectations, enhancing long-term engagement and collaboration in HRI.

## Introduction

Rapid advances in artificial intelligence, exemplified by technologies like GPT models^1^, are driving the societal integration of communication robots^2–4^. These robots are expected to be more than just high-performing; they must be perceived by humans as social partners^5^. A significant body of research shows that humans, in some cases, apply social rules and expectations to machines, treating them as social actors^6–8^. Nass and Moon (2000) demonstrated that people unknowingly respond to machines with the same politeness, teamwork, and reciprocity norms that govern human-human interactions^9^.

Consequently, much Human-Robot Interaction (HRI) research has focused on measuring user impressions. Standardized instruments like the Godspeed Questionnaire assess traits such as anthropomorphism, animacy, and likability^10^, while the Robotic Social Attributes Scale (ROSAS) measures perceived warmth and competence^11^, and the NARS measures negative attitudes toward robots^12^. In addition to these instruments, multidimensional HRI evaluation scales such as the Human-Robot Interaction Evaluation Scale (HRIES) have been proposed to capture broader perceptual components of HRI^13^. These social-perception dimensions are also closely linked to trust development in HRI, alongside anthropomorphism-related cues^14^. Meta-analytic evidence further indicates that anthropomorphism and embodiment can systematically affect a wide range of both subjective and objective outcomes in social HRI^15^. These scales are invaluable for capturing immediate reactions to a robot’s attributes. However, they primarily evaluate the robot in isolation. This focus often overlooks the "relational structure," a multifaceted concept encompassing the division of responsibilities, the balance of mutual contributions, and the degree of shared goals that defines how humans and robots interact in real-world contexts, referring to social studies^16,17^. Recent work also indicates that people categorize human-robot relationships into distinct perceived types (e.g., professional or communicative), suggesting that relationship framing varies systematically across contexts and should be measured explicitly^18^.

Ignoring these underlying relational complexities can severely hinder long-term acceptance and the quality of human-robot collaboration. For instance, the ideal balance of contribution differs significantly between a robot viewed as a partner for household tasks and one intended for conversation. A mismatch between a user’s expectations and the robot’s actual behavior can lead to user dissatisfaction^19^. Therefore, understanding and aligning these relational structures is not merely an academic exercise but a critical step toward fostering sustainable and meaningful collaborations.

This relational perspective is supported by foundational HRI studies, which confirm the importance of matching a robot’s appearance and behavior to its designated role and task^6^. More recent work urges a critical examination of the potential benefits and ethical risks of human-robot relationships, arguing that the type of relationship (e.g., peer-like friend or tool-like servant) brings different consequences^20^. Related findings further suggest that social connectedness can shape links between robot anthropomorphism and human dehumanization, underscoring the need for careful relationship framing^21^. Interpersonal relationship theories have been applied to human-robot relationships, yet there are important reasons to question whether such theories transfer straightforwardly to humanoid social robots and their interaction dynamics^22^. Our approach is deeply grounded in social psychology, particularly Fiske’s Relational Models Theory, which identifies universal frameworks like Communal Sharing, Authority Ranking, Equality Matching, and Market Pricing that organize social life^16,17^.

Tschopp et al. surveyed users of voice assistants and found that people tend to default to an “authority ranking” relationship with AI, essentially viewing the AI as a subordinate servant by default^23^. This aligns with findings that some people imagine domestic robots in utilitarian roles rather than as social companions^20^. Nevertheless, a study of household robots (e.g., Roomba) reported that participants develop an intimate connection with the device, deriving greater pleasure from cleaning, making efforts to integrate the robot into their homes, and even sharing it with others^24^. This demonstrates the human readiness to anthropomorphize and form social connections, reinforcing the importance of our research focus.

This study moves beyond simple impressions to focus explicitly on the "relational structure between humans and robots." We examine these structures from the perspective of mutual contributions and shared goals, concepts drawn from social psychology and game theory. To achieve this, we developed and validated a novel pictogram-based questionnaire designed to capture these complex relational dynamics intuitively, a methodological advancement in HRI. Our key finding is that a robot’s communicative utterances are more influential than its physical appearance in shaping users’ assumptions about the relational structure. By assessing these deep-seated perceptions, our work provides critical insights for designing robots capable of more effective, appropriate, and sustainable collaboration.

## Materials and methods

In this section, we describe the experiment we conducted to illustrate why it is crucial to consider structural patterns of relationships between humans and robots. As a preliminary step, we developed a questionnaire that uses pictograms to represent different types of social relations and measures how participants intuitively perceive these relationships. We also adopted the questionnaire for three online surveys.

### Questionnaire development

**Fig. 1:**
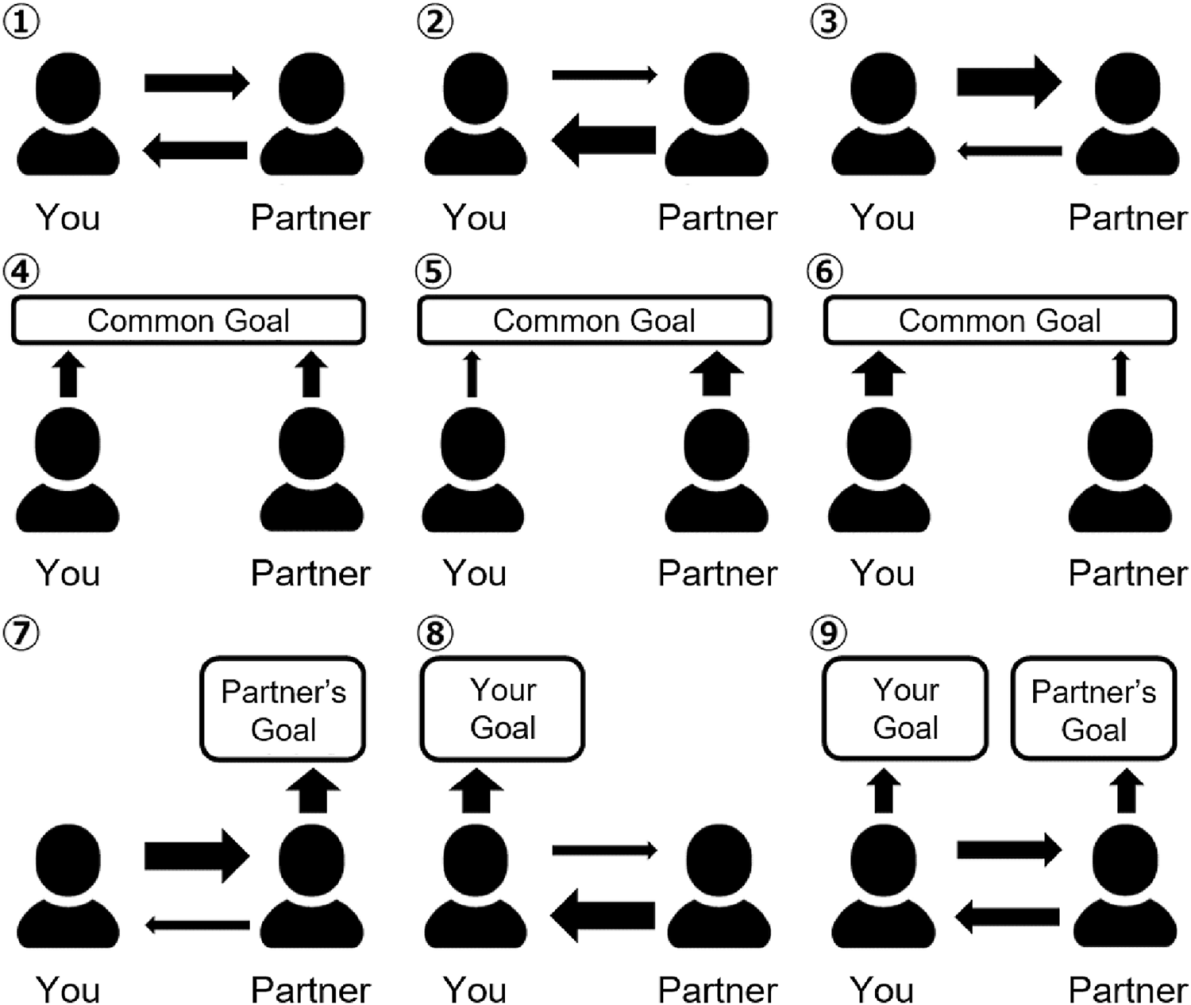
Pictograms representing nine relational structures. Each arrow denotes a direction of contribution (from the contributor to the recipient), and arrow thickness encodes the magnitude of contribution (thicker arrows indicate larger contributions). Structures $\textcircled {1}$–$\textcircled {3}$ depict direct reciprocity with balanced vs. imbalanced exchange; Structures $\textcircled {4}$–$\textcircled {6}$ represent contributions toward a shared goal; Structures $\textcircled {7}$–$\textcircled {9}$ illustrate goal-asymmetric or separate-goal relations with mutual influence. See the Methods section for detailed theoretical underpinnings. The pictogram’s original Japanese texts have been translated into English.

We prepared visual pictograms representing various relationship types to enable intuitive judgments and developed a questionnaire in which participants selected the most applicable pictogram. We developed the questionnaire to investigate relational structures, centering on two main dimensions: (i) each party’s contribution to the other, and (ii) the purpose or goals of each party. Specifically, we measured how people perceive their current relationship with a counterpart across scenarios and what they consider the ideal relational structure. We compiled various potential relation structures between humans and robots and asked participants to select the structure (represented by a pictogram) that best matched their perception. Finally, we created nine pictograms (Figure 1), primarily inspired by theoretical perspectives from social science studies as follows:

- Pictograms $\textcircled {1}$- $\textcircled {6}$ were based on reciprocity theory, which includes direct and indirect reciprocity.
  - One-to-one reciprocal relationship: it centers on whether two parties provide balanced contributions to each other (Reciprocal Altruism^25^). This can be seen in friendships where help is exchanged, or fair exchanges in business transactions. We thus derived the following:
    - Pictogram $\textcircled {1}$: Both parties equally contribute to each other: a balanced, ideal form of direct reciprocity.
    - Pictogram $\textcircled {2}$: The respondent receives more than they contribute: an arrangement where one party predominantly provides benefits to the other.
    - Pictogram $\textcircled {3}$: The respondent contributes more than they receive: imbalanced direct reciprocity that may occur under the assumption of future reciprocation.
  - Beyond one-to-one reciprocal relationships, public goods games consider benefits shared by a group, evaluating participants based on the extent of their contributions to the collectively beneficial public good. Reflecting this, we created:
    - Pictogram $\textcircled {4}$: Both parties contribute equally toward a shared goal (e.g., group volunteer activities).
    - Pictogram $\textcircled {5}$: An unequal distribution of contributions, as in a team where a leader shoulders heavier responsibility than do team members.
    - Pictogram $\textcircled {6}$: One party takes a leading role, and the other party supports that goal, exemplifying a relationship in which roles are clearly defined to achieve a common objective.
- Pictograms $\textcircled {7}$ and $\textcircled {8}$ capture relationships (e.g., caregiving) that may not be fully explained through reciprocity alone. A relationship that primarily achieves the goals of one party is called a communal relationship^26^: pictogram $\textcircled {7}$ represents a focus on the other party’s goal, while pictogram $\textcircled {8}$ represents a focus on one’s own goal.
- Pictogram $\textcircled {9}$ depicts a situation where both parties maintain separate goals but still exert positive, mutual influence. This arrangement aligns with theories related to complementarity^27^, evident in scenarios such as spouses who support each other’s careers. Here, the relationship is marked by sustainable cooperation that preserves each party’s independence.

### Assessment for developed questionnaire

Using these nine pictograms (see Figure 1), we constructed a questionnaire in which participants were presented with a scenario featuring a robot (e.g., its appearance or the interaction context) and asked to select the pictogram that best reflected the relationship structure they perceived or desired. We employed this questionnaire in the following surveys.

The pictograms were created through discussions among the authors. While no formal preliminary experiments were conducted to verify whether the authors’ intended meanings were accurately conveyed, the pictograms were shown in advance to multiple individuals, including members of the authors’ laboratory and students in their classes. After confirming that the pictograms created with the intended meanings were understood without issue, the surveys were conducted. Below, we detail how the developed questionnaire was assessed through these surveys.

### Survey A: ideal relationships in human-human contexts

In Survey A, participants first considered various human relationships (e.g., with a spouse, a friend, or a parent) and identified which of the nine pictograms (Figure 1) best represented their ideal relationship with that person. For instance, the question was: “Among the nine relationship patterns as shown, which one (1–9) best captures the ideal relationship between you and your [X] (where X is parent, spouse, friend, etc.)?”

### Survey B: ideal relationships in specific scenarios (Human vs. Robot)

Survey B examined how participants envision ideal relationships with humans or robots across specific scenarios (i.e., cleaning, counseling). We manipulated the following two factors to define the interaction context:

- Role (2 types)
  - Cleaning: An agent performs a non-dialogue task, here cleaning a room.
  - Counseling: An agent engages in a dialogue task, listening to the other’s concerns.
- Appearance (3 types)
  - Human
  - Human-like robot (Android)
  - Machine-like robot

**Fig. 2:**
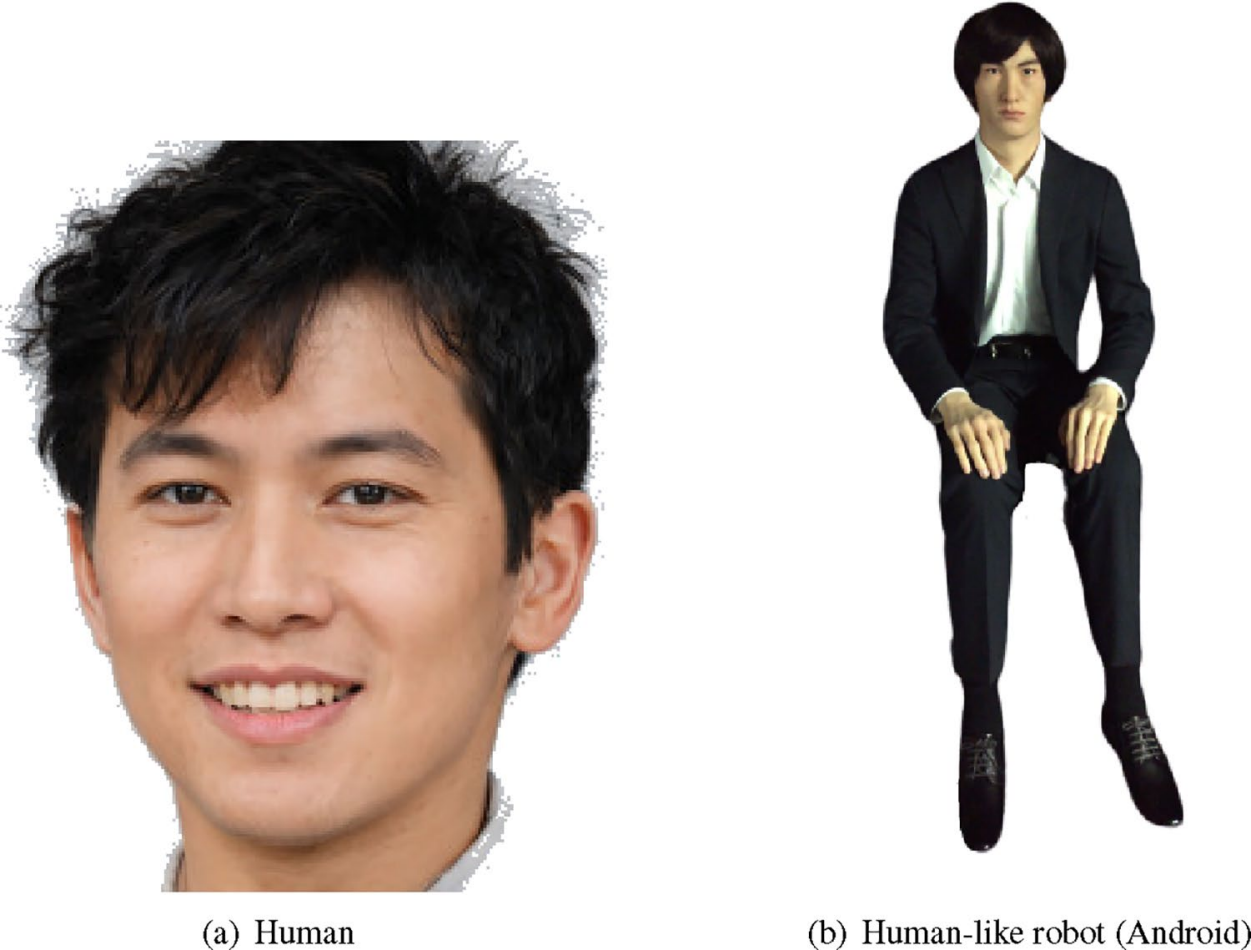
Appearances of the agents. The human image was generated by a photo generation tool (https://generated.photos/).

Each combination of Robot’s Role $\times$ Appearance was presented, and participants were asked to choose from the nine pictograms to indicate the most ideal relationship structure in that scenario. The appearance manipulations included a human (Figure 2 (a)), a human-like android(Figure 2 (b)), and a purely mechanical robot (Figure 2 (a) in a previous study^28^). In total, participants saw six scenarios (2 roles $\times$ 3 appearances) and were asked which pictogram best illustrated their ideal relationship in each.

### Survey C: assumed relationships in specific scenarios (Human vs. Robot)

Survey C investigated how the appearance and behavior of a human or robot counterpart influence the relationship structure participants expect to develop. Similar to Survey B, we combined three factors:

- Role (2 types)
  - Cleaning: An agent performs a non-dialogue task, here cleaning a room.
  - Counseling: An agent engages in a dialogue task, listening to the other’s concerns.
- Appearance (3 types)
  - Human
  - Human-like robot (Android)
  - Machine-like robot
- Utterance (3 types)
  - Service-Oriented Utterance: For example, “I will do anything to help you.” In this case, the agent’s contribution is relatively high, with minimal expectation of return from the other.
  - Reciprocity-Oriented Utterance: For example, “I enjoy listening to people’s problems, so please share your concerns with me,” or “I’d be happy if you praise me when I finish cleaning.” Here, the agent expects some form of return or positive feedback.
  - Goal-Oriented Utterance: For example, “I can handle certain parts of cleaning, but I need your help with the more difficult tasks,” anticipating a balanced exchange of contributions.

In total, participants were presented with nine scenarios for each robot role (3 appearances $\times$ 3 utterances = 9). They were asked to select which of the nine pictograms best captured the current relationship structure they believed would form under those conditions. Through these surveys, we aimed to elucidate how people conceptualize ideal and actual relational structures both in human-human and human-robot interactions based on mutual contributions and shared or individual goals. By comparing the structures that emerge across different roles, appearances, and behaviors, we hope to highlight the importance of relational structure in designing robots for long-term, meaningful collaboration.

### Psychometric evaluation of the pictogram instrument

Because the pictogram response is a forced-choice nominal selection (one of nine pictograms) rather than a set of additive questionnaire items, classical internal-consistency indices (e.g., Cronbach’s $\alpha$) are not directly applicable. We therefore evaluated reliability as within-participant *choice stability* across repeated trials and examined construct validity via theoretically-derived dimensions encoded in the pictograms.

#### Reliability (choice stability)

Within each utterance condition, each participant completed 6 trials (2 roles $\times$ 3 appearances). For each participant and each utterance type, we computed stability as the proportion of the modal response across the 6 trials (range: $1/6=0.17$ to 1.00). We report stability for the raw 9-category pictogram choice and for two derived construct dimensions encoded in the pictograms: Direction (3 categories; relative arrow magnitude/direction) and Goal structure (5 categories; goal alignment), as shown in Table 1. We deterministically mapped the nine pictograms into two categorical dimensions derived from the graphical semantics. First, *Direction* captured the relative direction/magnitude of contribution between the respondent and the partner based on arrow direction and thickness: *Equal* (pictograms $\textcircled {1}$, $\textcircled {4}$, and $\textcircled {9}$), *Partner > Respondent* (pictograms $\textcircled {2}$, $\textcircled {5}$, and $\textcircled {8}$), and *Respondent > Partner* (pictograms $\textcircled {3}$, $\textcircled {6}$, and $\textcircled {7}$). Second, *Goal structure* captured goal alignment based on whether contributions targeted a shared goal versus one agent’s goal: *Direct reciprocity* (pictograms $\textcircled {1}$–$\textcircled {3}$), *Common goal* (pictograms $\textcircled {4}$–$\textcircled {6}$), *Partner’s goal* (pictogram $\textcircled {7}$), *Your goal* (pictogram $\textcircled {8}$), and *Separate goals* (pictogram $\textcircled {9}$). These mappings are theory-driven and based on the intended semantics of the pictogram elements (arrow direction/thickness and goal frames), rather than data-driven clustering.

**Table 1:** Theory-driven mapping from nine pictograms to derived construct dimensions.

Pictogram	Direction (3-level)	Goal structure (5-level)
$\textcircled {1}$	Equal	Direct reciprocity
$\textcircled {2}$	Partner > Respondent	Direct reciprocity
$\textcircled {3}$	Respondent > Partner	Direct reciprocity
$\textcircled {4}$	Equal	Common goal
$\textcircled {5}$	Partner > Respondent	Common goal
$\textcircled {6}$	Respondent > Partner	Common goal
$\textcircled {7}$	Respondent > Partner	Partner’s goal
$\textcircled {8}$	Partner > Respondent	Respondent goal
$\textcircled {9}$	Equal	Separate goals

#### Construct validity

Each pictogram was mapped onto Direction (3-category) and Goal structure (5-category) constructs reflecting the intended semantics of arrow direction/magnitude and goal alignment cues. We tested whether utterance type systematically shifted these constructs using multinomial generalized linear mixed models with random intercepts for participants and fixed effects of role, appearance, and utterance.

### Statistical analysis

We first summarized pictogram-choice distributions in the ideal relationships scenario (Survey A and B) using chi-square tests and reported adjusted residuals to describe over- and under-selected pictograms.

To quantify the relative contributions of multiple predictors while accounting for the repeated-measures structure (each participant responded to all Survey C scenarios), we fitted a multinomial generalized linear mixed-effects model (GLMM). The dependent variable was the selected pictogram (nine nominal categories). Role (cleaning vs. counseling), appearance (human, android, machine-like robot), utterance (service-, reciprocity-, goal-oriented), age, and gender were included as fixed effects, and participant ID was included as a random intercept. Candidate models with interaction terms among role, appearance, and utterance were compared using information criteria, and we report the most parsimonious model with the best fit.

## Results

### Participants

Each participant evaluated all scenarios and all relationships in the questionnaire. To counterbalance potential order effects, we randomized both the order of the robot’s role/appearance combinations and the order in which each behavior was presented. We recruited participants via Yahoo Crowdsourcing, yielding a sample of 510 individuals (279 males, 227 females, and 4 others; mean age = 37.04, SD = 6.64). They responded to a web-based platform. All participants provided informed consent before the start of the experiment, which was approved by the Ethics Committee of Osaka University, Japan. We confirmed that all the experiments were performed in accordance with relevant guidelines and regulations.

### Survey A: ideal relationships in human-human contexts

A chi-square test confirmed a significant effect ($\chi ^{2}(32) = 411.298, p <.01$). The results of the residual analysis are shown in Table 2. The residual analysis indicated that relationships $\textcircled {2}$ and $\textcircled {8}$ were selected significantly more often than expected for relationships with parents; relationship $\textcircled {4}$ for relationships with spouses and lovers; relationship $\textcircled {1}$ for friendships; and relationships $\textcircled {3}$ and $\textcircled {7}$ for relationships with children.

**Table 2:** Adjusted residuals from the chi-square analysis for ideal relationship structures (Pictograms $\textcircled {1}$- $\textcircled {9}$) in different human-human relationships (Survey A). Positive values indicate significantly higher selection frequency than expected, while negative values indicate significantly lower frequency. **: $p < .01$, *: $p < .05$, +: $p <.10$.

Relation	1	2	3	4	5	6	7	8	9
Parent	−0.76	5.56**	0.00	−3.56**	1.07	0.22	−2.44*	4.54**	−0.09
Spouse	−0.56	−2.76**	−3.92**	6.28**	−0.36	−0.88	−3.17**	−1.14	1.30
Lover	1.61	0.09	−3.13**	2.02*	−1.07	−0.88	−2.92**	−1.70+	0.95
Friend	5.64**	−2.10*	−4.11**	−0.37	1.07	−0.15	−3.41**	−1.42	−0.56
Child	−5.93**	−0.79	11.16**	−4.36**	−0.71	1.69+	11.94**	−0.28	−1.60

### Survey B: ideal relationships in specific scenarios (Human vs. Robot)

For each role, we tested whether the ideal relationship structure differed by the partner’s appearance. First, when the partner has the role of cleaning, a chi-square test was performed for the appearance and the structure of the nine relationships ($\chi ^{2}(16) = 34.840, p <.01$), and a residual analysis was performed (Table 3). As a result, relationship $\textcircled {1}$ was selected as ideal when the partner is human, and relationships $\textcircled {5}$ and $\textcircled {8}$ were selected as ideal when the robot has a machine-like appearance. On the other hand, when the robot had a human-like appearance, no relationship structure was significantly selected as ideal. The same chi-square test was conducted when the robot had the role of a counselor. The results showed that no relationship structure was significantly selected as ideal for either partner’s appearance ($\chi ^{2}(16) = 19.272, n.s.$).

**Table 3:** Adjusted residuals from the chi-square analysis of ideal relationship structures (Pictograms $\textcircled {1}$- $\textcircled {9}$) across partner appearances in Survey B. Positive values indicate higher-than-expected selection frequency, whereas negative values indicate lower-than-expected frequency. **: $p < .01$, *: $p < .05$, +: $p < .10$.

Relation	1	2	3	4	5	6	7	8	9
Human	3.63**	−0.46	−1.03	1.86+	−2.73**	0.00	1.04	−2.58**	0.70
Android	−1.66+	1.54	0.00	−1.01	0.67	1.38	−0.38	0.54	−0.65
Machine-like robot	−1.98*	−1.08	1.03	−0.85	2.06*	−1.38	−0.66	2.03*	−0.05

### Survey C: assumed relationships in specific scenarios (Human vs. Robot)

#### Multinomial GLMM analysis

To model pictogram choice while accounting for repeated responses within participants, we fitted a multinomial GLMM with a random intercept for participant ID. The age was mean-centered, and the gender was operationalized as a binary (responses outside these categories (i.e., four participants) were not included in the GLMM analyses) to facilitate interpretation of main effects in the presence of interaction terms (e.g., age$\times$utterance). The fixed-effects tests indicated significant main effects of role and utterance, whereas appearance was not significant when modeled jointly with other factors (Table 4). Notably, comparing the two three-level manipulated factors (utterance and appearance), utterance showed markedly stronger evidence of an effect on pictogram choice (larger *F*), whereas appearance was not significant.

**Table 4:** Type III tests of fixed effects in the multinomial GLMM for Survey C.

Predictor	*F*	df$_1$	df$_2$	*p*
Role	7.140	8	9044	$<.001$
Appearance	0.606	16	9044	.882
Utterance	110.026	16	9044	$<.001$
Age	2.451	8	9044	.012
Gender	2.755	8	9044	.005

We compared a set of multinomial mixed-effects models with and without two-way interactions using information criteria. The main-effects model (Model0) provided the best fit (lowest AICc/BIC), and adding interaction terms did not improve model fit (see Table 5).

**Table 5:** Model comparison for multinomial mixed-effects models predicting pictogram choice (Survey C). All models include a random intercept for participant ID. Lower AICc/BIC indicates better fit.

Model	Fixed effects	$-2LL$	AICc	BIC
Model0	role + appearance + utterance + age + gender	298400.412	298416.428	298473.291
Model1	Model0 + appearance$\times$utterance	298598.877	298614.893	298671.728
Model2	Model0 + appearance$\times$utterance + role$\times$utterance	299310.823	299326.839	299383.659
Model3	Model0 + appearance$\times$utterance + role$\times$appearance	301998.809	302014.825	302071.646
Model4	Model0 + appearance$\times$utterance + role$\times$utterance + role$\times$appearance	302713.094	302729.110	302785.916

#### Demographic moderation

To examine whether demographic variables moderate the effect of utterance on pictogram choice in Survey C, we extended the multinomial GLMM (random intercept for participant ID) by adding two prespecified interaction terms: age $\times$ utterance and gender $\times$ utterance. Both interactions were statistically significant in the Type III tests (age $\times$ utterance: $F(16,9012)=1.690$, $p=.041$; gender $\times$ utterance: $F(16,9012)=2.043$, $p=.008$), indicating that the association between utterance type and pictogram choice varies as a function of age and gender. Main effects of age and gender were also significant in this model (age: $F(8,9012)=2.342$, $p=.016$; gender: $F(8,9012)=2.244$, $p=.022$).

Importantly, including these demographic moderation terms did not alter the main pattern of results regarding the experimentally manipulated factors: utterance remained a robust predictor of pictogram choice ($F(16,9012)=107.366$, $p<.001$), whereas appearance remained non-significant ($F(16,9012)=0.607$, $p=.881$). This is consistent with the primary main-effects model (Model0).

### Reliability and construct validity of the pictogram instrument

For reliability, we computed choice stability within each utterance condition as the proportion of the modal response across the 6 trials (2 roles $\times$ 3 appearances) per participant (Table 6). Stability was moderate for the raw 9-category pictogram choice ($M = 0.517-0.636$ across utterance types) and higher for the two theoretically-derived dimensions: Direction (relative arrow magnitude/direction; $M = 0.681-0.799$) and Goal structure (goal alignment; $M = 0.632-0.727$). These results suggest that participants’ responses were not random but showed structured within-participant consistency, and that the derived dimensions (Direction and Goal structure) captured the intended semantics of key visual cues (arrow direction/magnitude and goal framing).

For construct validity, we mapped the nine pictograms onto the intended theoretical constructs (Direction and Goal structure) and tested whether the experimental manipulation (utterance type) systematically shifted these constructs using multinomial generalized linear mixed models (GLMM) with random intercepts for participants. Utterance type had a robust effect on both Direction ($F(4,9168)=225.843, p<.001$) and Goal structure ($F(8,9156)=180.744, p<.001$), whereas appearance did not (Direction: $F(4,9168)=1.192, p=.312$; Goal structure: $F(8,9156)=0.408, p=.916$). These results suggest that the utterance type robustly predicted both Direction and Goal structure, whereas appearance did not, supporting construct validity of the visual cues.

**Table 6:** Within-participant choice stability (mean [95% CI]) by utterance type. Stability is the proportion of the modal response across 6 trials (2 roles $\times$ 3 appearances) within each utterance condition.

	Service-oriented	Reciprocity-oriented	Goal-oriented
Raw pictogram (9 categories)	0.517 [0.502, 0.533]	0.561 [0.542, 0.579]	0.636 [0.617, 0.655]
Direction (3 categories)	0.681 [0.665, 0.697]	0.695 [0.679, 0.712]	0.799 [0.782, 0.816]
Goal structure (5 categories)	0.632 [0.615, 0.650]	0.649 [0.630, 0.667]	0.727 [0.709, 0.744]

## Discussion

This study enhances our understanding of HRI by shifting the evaluation focus from traditional impression-based measures (e.g., anthropomorphism and likability) to an analysis of the perceived relational structure between humans and robots. Specifically, we proposed and validated a novel questionnaire that captures relational dimensions such as mutual contributions, division of responsibilities, and shared goals, consistent with social psychological theory.

Survey A revealed that ideal relational structures differ based on specific human relationships. For instance, balanced reciprocal relationships were preferred in friendships, whereas communal or caregiving relationships were favored in parent-child dynamics.

Survey B showed that appearance significantly influenced ideal relational structures primarily in task-specific contexts, such as cleaning. Participants favored balanced reciprocity with human partners, whereas clearly defined roles and service-oriented relationships were preferred with mechanical robots. Human-like robots elicited ambiguous relational expectations, indicating that designers should ensure robots clearly communicate their relational intentions. Prior work also suggests that role-taking processes and robotic form can shape perceived social connection in HRI^29^, consistent with the appearance-driven shifts observed in our Survey B. Notably, no single ideal relational structure was significantly preferred for the human-like android in the cleaning scenario. We interpret this as indicating heterogeneous framing: an android appearance can elicit competing schemas (human-like partner vs. engineered tool), leading different participants to prefer different relational models and resulting in a dispersed distribution. This ambiguity suggests that human-like appearance alone may be insufficient to communicate relational intent; designers may need stronger contextual framing and/or consistent communicative cues to stabilize users’ expectations.

Survey C clarified how robot utterance framing affects assumed relational perceptions. In the multinomial GLMM, utterance showed a robust association with pictogram choice, whereas appearance was not significant when modeled jointly with other factors (Table 4). Consistent with this, utterance also robustly shifted the two theoretically-derived constructs encoded in the pictograms (Direction and Goal structure), supporting the role of communicative framing as a key design cue for shaping perceived contribution balance and goal alignment. This is consistent with evidence from strategic interaction paradigms showing that how a robot communicates can influence cooperation with human partners^30^. Thus, a robot’s communication style is crucial in shaping perceptions of relational fairness and goal sharing. Such relational expectations may also extend to downstream prosocial behaviors toward robots (e.g., willingness to help), which can depend on attitudes or the context of use^31^. Although we operationalized role using cleaning (non-dialogue task) and counseling (dialogue task), the underlying dimensions of contribution balance and goal alignment are not domain-specific. In healthcare or caregiving contexts, for example, a communal/care-oriented relational structure may be expected but must be designed to protect user autonomy. In industrial or high-stakes team settings, shared-goal cooperation and clearly negotiated responsibilities may be more appropriate, and misalignment could have larger practical consequences. Expanding scenarios to these domains is an important direction for future validation.

These findings suggest the desirability of robots capable of adapting to varied relational expectations. Dautenhahn et al. (2005) surveyed adults about future home robot companions and found a majority envisioned the robot as an assistant, machine, or servant, rather than as a social friend^32^. Participants prioritized practical help with household tasks over companionship, and only a few wanted a human-like friend. Additionally, human-like communication was appreciated, but a human-like appearance was seen as less important. These results resonate with our Survey A findings: participants’ “ideal” relational structures with a robot at home skewed toward clearly defined service or helper roles, rather than peer friendship. It underlines that the desired human-robot relationship can be context-dependent; in domestic settings, utility may trump intimacy.

Compared with prior work, our contributions are threefold. First, we systematically focused on multi-dimensional relational structures rather than on simple, robot-specific impressions. Second, we introduced a unique approach combining insights from psychological relationship theories (e.g., reciprocity, communal relationships, and complementarity) and game theory (i.e., payoff relationships). This interdisciplinary approach allowed us to conceptualize and measure human-robot relationships comprehensively. Lastly, our innovative use of intuitive pictograms as measurement tools facilitated participants’ understanding and evaluation of complex relational dynamics, which represents a methodological advancement within HRI studies.

Our findings have implications for long-term human-robot engagement. Prior research on relational agents (virtual humans designed to build relationships) shows that when an agent behaves in ways that maintain a social bond over time, users come to like and trust it more, and are more willing to continue the interaction. Bickmore and Picard (2005) demonstrated this in a month-long study: participants who interacted daily with a relational agent that used socio-emotional cues (like remembering personal details, exhibiting empathy) reported significantly greater trust and desire to keep using the agent compared with a task-only agent^33^. In HRI, this suggests that robots aligning with the user’s expected relational model (e.g., a collaborative partner showing understanding, or a service robot reliably fulfilling requests) could foster better long-term outcomes (higher satisfaction, trust, and sustained use) than robots that violate those expectations. Thus, designing robots with an eye toward the intended relationship dynamic (not just functionality) is key for enduring human-robot relationships.

Importantly, real-world case studies of home robot adoption show the dynamic nature of human-robot relationship formation. In a field study, De Graaf et al. (2015) introduced a social robot (“Harvey”) into households for several weeks and observed how users’ attitudes and relationship perceptions changed^34^. Initially, participants tended to treat the robot cautiously as a novel gadget, but over time, many started assigning it a social role: some saw it as a kind of pet or companion providing comfort, while others kept viewing it as a useful household appliance. Crucially, users who reported a stronger emotional connection (naming the robot, feeling responsible for it) were more likely to continue using the robot regularly. This resonates with our finding that perceived relational structure (e.g., companion vs. tool) can impact long-term acceptance. It suggests that facilitating the desired relational structure (for instance, through robot behavior that encourages either companionship or efficient service as appropriate) could be a strategy to improve user satisfaction and sustained use.

Different perceived relational structures can entail distinct ethical risks. A strongly service/servant-like framing may reinforce power asymmetries and encourage overdelegation, whereas a friend-like framing can increase emotional reliance and may inflate trust beyond a system’s actual capabilities. Miscalibrated relational trust can in turn affect user autonomy and decision-making in the long run. A practical value of measuring relational structure is that it enables designers and stakeholders to audit whether specific appearance/utterance choices systematically steer users toward potentially problematic relationship models, supporting more responsible relationship framing.

The relational structure a user assigns to a robot is unlikely to be static. Early encounters may be shaped by novelty, uncertainty, and role/appearance cues, whereas repeated interaction can shift expectations toward patterns grounded in reliability, accumulated reciprocity, and shared routines. For example, a robot initially perceived as a one-sided service provider may be reframed as a cooperative partner if it consistently negotiates shared goals and responds contingently to the user’s contributions. Conversely, trust violations or misaligned communication may push perceptions toward more transactional or avoidant frames. Because the proposed instrument is lightweight, it can be administered repeatedly (e.g., after each session) to characterize these trajectories and to identify when design interventions (e.g., changes in utterance style) alter the perceived relationship model.

However, several limitations must be acknowledged. Firstly, our study relied exclusively on self-report questionnaires based on hypothetical scenarios. As a result, the measured ‘ideal’ and ‘assumed’ relational structures should be interpreted as participants’ subjective projections under the described conditions. Such projected perceptions may align with, but can also differ from, the relational dynamics and behavioral responses that emerge during real-time, embodied human-robot interaction (e.g., effects of physical presence, interaction timing, and contingent responses). Therefore, future research should validate these findings by incorporating direct interactions with physical robots and observational/behavioral measures of interaction (e.g., cooperation, turn-taking, or helping behavior) alongside repeated pictogram assessments.

Secondly, our investigation primarily evaluated initial or hypothetical perceptions of relationships, without considering how relational structures evolve over prolonged interactions. Longitudinal studies are necessary to understand dynamic changes and stability in human-robot relationships. Thirdly, the generalizability of our results might be constrained by cultural homogeneity, considering robot impressions (e.g., anthropomorphism^35^) can be modulated by culture.

Our participants were recruited via Yahoo Crowdsourcing in Japan, resulting in a culturally and linguistically homogeneous sample. Given that relationship norms and robot-related expectations can vary across cultures, the observed distributions and the relative salience of appearance vs. utterance may not generalize. Future work should replicate the study across multiple countries/languages and evaluate cross-cultural measurement invariance (i.e., whether the visual encodings and category meanings are interpreted equivalently).

Additionally, while pictograms offered an intuitive evaluation method, they may oversimplify complex relationship dynamics. Supplementary qualitative methods, such as interviews, could help uncover relational subtleties not captured by pictograms alone.

Furthermore, our research scenarios were limited to specific contexts (e.g., cleaning and counseling). Expanding scenarios to encompass a broader range of interaction settings, such as education, healthcare, or entertainment, could enhance the applicability and robustness of our relational model.

The questionnaire required a single selection among nine relational structures and did not provide a ‘no relationship/avoidance’ option. Participants who preferred disengagement may therefore have selected the closest available category, potentially biasing the observed distributions. Future versions should include an explicit avoidance option (e.g., a ‘no relationship’ response or an additional pictogram) and/or allow respondents to indicate that none of the nine structures apply.

Finally, while we found that demographic factors can moderate how utterance types map onto perceived relational structures (significant age$\times$utterance and gender$\times$utterance interactions), adding these moderation terms did not alter the main pattern of results: utterance remained robustly associated with pictogram choice whereas appearance remained non-significant in the joint model. Our analyses do not fully resolve whether such demographic tendencies are robot-specific (i.e., stronger for robots than for humans). Addressing this interpretation would require explicit tests of demographic interactions with appearance (e.g., age $\times$ appearance) and/or targeted comparisons between human and robot counterparts, which we leave for future work. Accordingly, we interpret the present results as a first-step mapping from design cues to expected relational models, and we avoid claims about real-time interaction outcomes without behavioral validation.

In conclusion, our research presents a significant advancement in understanding the complexities of human-robot relational structures through a comprehensive, psychologically grounded, and intuitively accessible measurement approach. Despite its limitations, the developed questionnaire and the obtained insights contribute valuable perspectives to HRI research, guiding future investigations toward fostering meaningful, effective, and sustainable human-robot collaborations.

## Data Availability

The data in this study are provided within the manuscript.
